# The Relationship between Military Combat and Cardiovascular Risk: A Systematic Review and Meta-Analysis

**DOI:** 10.1155/2019/9849465

**Published:** 2019-12-22

**Authors:** Christopher J. Boos, Norman De Villiers, Daniel Dyball, Alison McConnell, Alexander N. Bennett

**Affiliations:** ^1^Academic Department of Military Rehabilitation, Defence Medical Rehabilitation Centre, Near Loughborough, Stanford Hall Estate, Nottinghamshire LE12 5QW, UK; ^2^King's Centre for Military Health Research, King's College London, UK; ^3^Faculty of Health & Social Sciences, Bournemouth University, Bournemouth BH1 3LT, UK; ^4^Department of Cardiology, Poole Hospital NHS Foundation Trust, Poole BH15 2JB, UK; ^5^National Heart and Lung Institute, Faculty of Medicine, Imperial College London, UK

## Abstract

**Background and Objectives:**

Cardiovascular disease (CVD) is a leading cause of death among military veterans with several reports suggesting a link between combat and related traumatic injury (TI) to an increased CVD risk. The aim of this paper is to conduct a widespread systematic review and meta-analysis of the relationship between military combat ± TI to CVD and its associated risk factors.

**Methods:**

PubMed, EmbaseProQuest, Cinahl databases and Cochrane Reviews were examined for all published observational studies (any language) reporting on CVD risk and outcomes, following military combat exposure ± TI versus a comparative nonexposed control population. Two investigators independently extracted data. Data quality was rated and rated using the 20-item AXIS Critical Appraisal Tool. The risk of bias (ROB using the ROBANS 6 item tool) and strength of evidence (SOE) were also critically appraised.

**Results:**

From 4499 citations, 26 studies (14 cross sectional and 12 cohort; 78–100% male) met the inclusion criteria. The follow up period ranged from 1 to 43.6 years with a sample size ranging from 19 to 621901 participants in the combat group. Combat-related TI was associated with a significantly increased risk for CVD (RR 1.80: 95% CI 1.24–2.62; *I*^2^ = 59%, *p* = 0.002) and coronary heart disease (CHD)-related death (risk ratio 1.57: 95% CI 1.35–1.83; *I*^2^ = 0%, *p* = 0.77: *p* < 0.0001), although the SOE was low. Military combat (without TI) was linked to a marginal, yet significantly lower pooled risk (low SOE) of cardiovascular death in the active combat versus control population (RR 0.90: CI 0.83–0.98; *I*^2^ = 47%, *p* = 0.02). There was insufficient evidence linking combat ± TI to any other cardiovascular outcomes or risk factors.

**Conclusion:**

There is low SOE to support a link between combat-related TI and both cardiovascular and CHD-related mortality. There is insufficient evidence to support a positive association between military combat ± any other adverse cardiovascular outcomes or risk factors. Data from well conducted prospective cohort studies following combat are needed.

## 1. Introduction

In 1979 US Veterans Administration published the results of their review examining the potential causal relationship between traumatic limb amputation and future risk of cardiovascular disease (CVD) [1]. As part of this work a literature review was undertaken to examine the medical literature relating to traumatic amputation and future CVD risk. Among the publications examined were just six studies [2–7] that had reported cardiovascular outcomes (including hypertension and cardiovascular death) following traumatic amputation. The results were inconsistent and failed to show a clear relationship.

Owing to the inconsistency of existing published data, coupled with their concern regarding the health implications of a potential link between increased CVD risk, combat-related amputations and potentially other forms of severe traumatic injury (TI), the Veteran's Administration concluded that more robust data was required. Consequently, the Veteran's Administration and Department of US Defence commissioned a longitudinal study to more robustly investigate the issue. This retrospective cohort study was the first to provide evidence to support a significant link between combat-related traumatic amputation and a higher risk of future adverse cardiovascular outcomes [8, 9]. Unfortunately, this data represented military populations who were injured more than seventy years ago, and the relevance for those injured in current conflicts is open to question. Subsequent to this, only one systematic review and one literature review have emerged. They were both published approximately 10 years ago, only identified a handful of additional studies and failed to reach a consensus opinion [10, 11].

Consequently, and in light of the high tempo and large scale of recent military conflicts, there is a need to re-examine the issue of combat related injury and CVD risk. Recent wars in Iraq and Afghanistan have led to the survival of large numbers of combatants, who have sustained highly complex and severe trauma, which would most likely have proved fatal as little as 20 years ago. Despite this, there has not been a wider examination of the impact of unselected combat on CVD outcome (e.g., cardiovascular death) and its associated risk factors (e.g., hypertension and lipid profiles).

The objective of this review was to systematically search and review the literature to determine whether military combat exposure, both with and separately without TI is linked to an increased CVD risk and adverse outcomes.

## 2. Methods

### 2.1. Search Strategy

We conducted a systematic review and meta-analysis according to a pre-defined protocol and in accordance to the Preferred Reporting Items for Systematic Reviews and Meta-Analyses (PRISMA) guidelines [12]. The protocol of this review was prospectively registered at PROSPERO. Four electronic databases were used: PubMed, Embase, Cumulative Index to Nursing and Allied Health Literature (CINAHL) and ProQuest. Cochrane Reviews was also searched to identify any previous systematic reviews. A systematic search was undertaken for articles published between the 1^st^ of January 1980 and 22^nd^ December 2018, in any language. Two reviewers (NDV and CJB) worked in conjunction with a Medical Librarian to create a search algorithm, which used Medical Subject Headings (MeSH) terms, where available.

The search was conducted in adherence to the PICO (Population, Intervention/exposure, Comparison/control and Outcome) tool [13]. The population search terms examined were (“Military”, “veterans”, “combat”, “servicemen”, “Iraq”, “Afghanistan”, “Army”, “armed services”, “marines” or “infantry”). The Intervention search terms used were (“traumatic”, “trauma-related”, “amputation”, “amputees”, “traumatic injury”, “wounded”, “wounding”, “combat”, “warfare”, or “battlefield”). The Outcome search terms were cardiovascular (including “cardiovascular death”, “cardiovascular event”, “cardiovascular mortality” and “cardiovascular risk” and related terms (“coronary heart disease (CHD)”, “ischemic heart disease”, “coronary artery disease”, “myocardial infarction”, “acute coronary syndrome”, “peripheral arterial disease”, “peripheral vascular disease”, “atrial fibrillation”, “arterial hypertension”, “high blood pressure”, “atrial fibrillation”, “heart failure”, “stroke”, “aortic aneurysm”, “coronary artery bypass” “coronary artery intervention”, “coronary artery stenting”, “diabetes mellitus”, “metabolic syndrome”, “carotid intimal thickness”, “augmentation index”, “arterial stiffness” or “pulse wave velocity” or “pulse waveform analysis”). The reference sections of eligible full-text articles were also examined to identify additional studies suitable for inclusion that might have been missed by the search algorithm.

### 2.2. Study Selection

Only observational studies that evaluated the impact of combat exposure ± TI on future cardiovascular outcomes were included. Individual case reports, conference abstracts, animal studies, *in vivo*/*in vitro* studies and those involving children, were excluded. Studies relating to starvation, cold injury or famine were excluded. Studies that examined selected groups of combatants with traumatic brain injury, spinal cord injury and post-traumatic stress disorder (PTSD) were also excluded.

All selected studies needed to include a population of currently serving, or ex-military and predominantly (>75%) male servicemen (veterans) who had been exposed to combat operations. A Comparator or Control group of nonexposed controls was required with a period of follow up from exposure to outcome of at least one year (see selection algorithm Supplement [Supplementary-material supplementary-material-1]).

**Figure 1:**
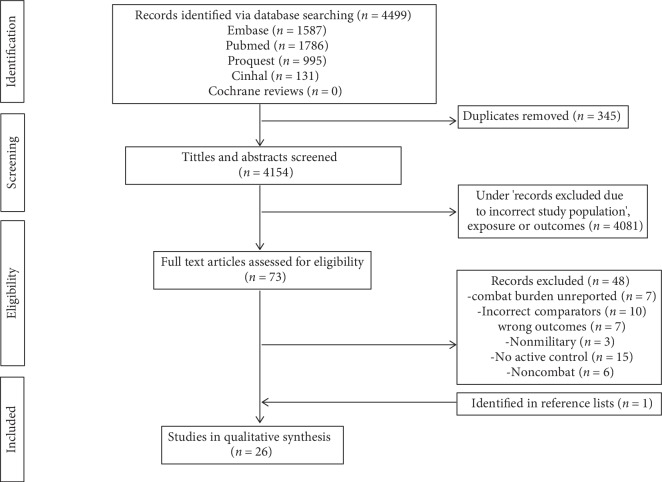
PRISMA flow diagram representing search and selection of studies.

Two reviewers (CJB and NDV) examined all of the screened records independently to determine potential for inclusion. For each study a preliminary grading of “include, exclude or unclear” was made. Study eligibility was assessed on the basis of the article title, followed by examination of the abstract. After the preliminary screening process, full text versions of articles deemed “included” and “unclear” were scrutinised further. Eligible studies were identified based on the inclusion criteria. Any disagreements between reviewers were resolved by a detailed discussion in order to come to a consensus. The PRISMA flowchart for the selection of included studies is shown in Figure 1.

### 2.3. Data Collection and Abstraction

Following selection, the data for each study was extracted using a pre-designed data extraction form, which included author, year of publication, military conflict and population studied, number of participants, type of study, sex, duration of follow up, study outcomes and findings (Table 1).

### 2.4. Quality Assessment

The AXIS Critical Appraisal tool was used to critically appraise the quality of each of the included studies [14] (Supplement [Supplementary-material supplementary-material-1]). The AXIS tool consists of 20 questions relating to study conduct. Studies with a total score of >15 were deemed to be high quality, those of 10–15 of moderate quality, whilst those scoring <10 were deemed poor quality. The Risk of Bias (ROB) of all included studies was examined using the Risk of Bias Assessment Tool for Nonrandomized Studies (RoBANS), which consists of six questions (Supplement [Supplementary-material supplementary-material-1]) [15]. Using the total scores for each study the ROB was graded as low (scores of 0), moderate (1-2) or high (>2).

### 2.5. Study Outcomes

The study outcomes were cardiovascular death, CHD-related death, CHD and myocardial infarction, arterial hypertension, atrial fibrillation, stroke, heart failure, aortic aneurysms, peripheral vascular disease, diabetes mellitus, metabolic syndrome, carotid intima medial thickness and measures of arterial stiffness (including augmentation index and pulse wave velocity).

### 2.6. Statistical Analysis

Due to the heterogeneity of the included studies (cross sectional and cohort designs, mode of data presentation, etc.) a pooled analysis was inappropriate for the majority of the reported data. Accordingly, a narrative synthesis was undertaken for most included studies. A meta-analysis was undertaken separately for the binary outcome of CVD and CHD-related death, as these outcomes were only reported from cohort studies. Data was pooled using a random-effects model and the Cochran–Mantel–Haenszel Estimate for the generation of weighted risk ratios and their 95% confidence intervals.

Analysis of continuous data was undertaken using GraphPad Prism® (version 6.07) with results presented as the mean ± standard deviation (SD). Meta-analyses were conducted using RevMan (Review Manager) software (Version 5.3). Heterogeneity was evaluated using forest plots and the *I*^2^ statistic; *I*^2^ values of 25%, 50%, and 75% were considered evidence of low, moderate, and high heterogeneity, respectively [16]. The overall strength of evidence (SOE) for the included studies was assessed using the five domains of consistency, precision, reporting bias, study limitations and directness, as supported by the Cochrane Collaboration Tool [17] . The overall SOE was rated by a single investigator (CJB) with a final rating of high, moderate, low, or insufficient as previously described [18].

## 3. Results

The initial search retrieved 4499 potentially relevant studies, from which 345 duplicates were removed immediately. Following the preliminary title and abstract review, 73 full-text articles were screened for eligibility. Forty eight full-text articles failed to fully meet the systemic review inclusion criteria and were excluded. One additional study, which met our selection criteria, was identified from the reference lists (Figure 1) and included. Hence, 26 studies were included in this review. Agreement between the two reviewers on the selection of full-text articles was moderate Cohens Cohen's *κ* 0.70 (86.3% agreement).

### 3.1. Study Characteristics

All of the included studies were observational, 14 being cross sectional and 12 being cohort studies (Table 1). The follow up period ranged from 1 to 43.6 years. The sample size of the combat exposed population ranged from 19 to 621901 participants. One study was published in French. Fourteen studies related to US military war veterans (Table 1); other represented populations included UK, Iranian, Israeli, German and Finnish veterans. The studies covered a total of eight major combat operations: World War I (1918) and II (1939–45), The Korean War (1950–53), Vietnam War (1961–1975), the Iran-Iraq Wars (1980–1988), Israeli Conflicts (1948–1974), Gulf War I (1991) and the recent US/UK operations in Iraq and Afghanistan (2003–2014). Twelve studies comprised of participants with combat-related TI, whilst 14 studies included combat veterans without a significant burden of TI (predominantly noninjured).

The age ranges at the time of study enrolment ranged from 18 to 89 years. The study populations were predominantly male (range 78–100%). The majority of participants were, where stated, Caucasian (62.4–100%), with the vast majority (≥74% of stated) being of nonofficer rank at the time of combat (±TI).

### 3.2. Study Quality and Risk of Bias

The quality scores for the 26 studies ranged from 6 to 19 (out of maximum of 20; see Supplementary [Supplementary-material supplementary-material-1]). The mean quality score for all studies was 12.6 ± 3.1. Five studies were considered high quality (scores >15), 16 moderate quality [10–15] and five low quality (<10) (see Supplementary [Supplementary-material supplementary-material-1]). The risk of bias was considered significant in 19/26 studies (73.1%) (see Supplementary [Supplementary-material supplementary-material-1]).

### 3.3. Cardiovascular Mortality

**Figure 2:**
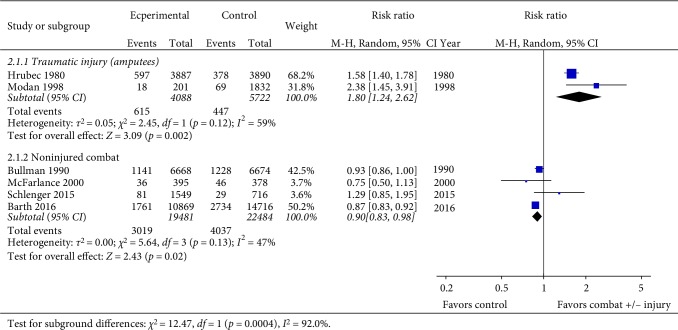
Pooled analysis of studies for the outcome of cardiovascular death.

The outcome of cardiovascular death (Tables 1 and 2) was reported in six cohort studies. Hrubec and Ryder [9] observed an increased risk of adjusted all-cause (low ROB; RR:1.36: 95% Confidence Interval (CI) 1.25–1.48), CVD (RR:1.58: CI 1.40–1.79) and CHD-related death (RR:1.56: CI 1.36–1.79) among proximal amputees at/above knee or elbow) vs. injured (wound or fractures without amputation) controls in their retrospective analysis of a ≥30 year follow-up of injured World War II veterans [9]. Modan (moderate ROB) reported a two-fold higher CVD mortality risk following a 24 year follow-up of 201 wounded Israeli veterans with unilateral lower limb amputations compared with a sample (*n* = 1832) of the general US population of similar age [19]. The pooled data (Figure 2) from these two studies demonstrated a significantly increased risk of cardiovascular related death (RR 1.80: 95% CI 1.24–2.62; *I*^2^ = 59%, *p* = 0.002). Meta-analysis of the four studies (one low, one moderate and two high ROB) investigating the effects of combat, without traumatic injury, identified a marginal, yet significantly lower pooled risk of cardiovascular death (hence no increase) in the active combat versus control population (RR 0.90: CI 0.83–0.98; *I*^2^ = 47%, *p* = 0.02) [20–23] (Figure 2).

### 3.4. Coronary Heart Disease (CHD) Mortality

**Figure 3:**
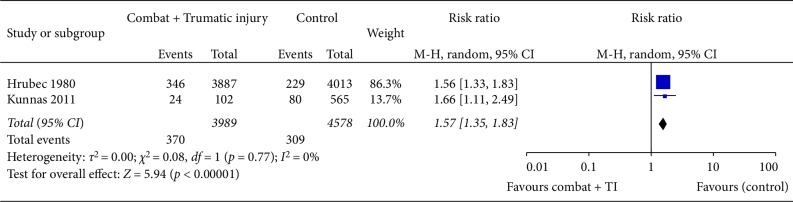
Pooled analysis of studies for the outcome of coronary heart disease death.

CHD-related death was reported in only three studies (Tables 1 and 2). Hrubec and Ryder observed higher adjusted CHD related death (low ROB; RR 1.56: CI 1.36–1.79) amongst combat veterans with traumatic proximal amputations versus controls with disfigurement injuries [9]. Kunnas et al. reported a higher adjusted risk of CHD (moderate ROB; RR 1.7: CI 1.1–2.5; *p* = 0.02) among injured combat veterans versus non-injured veterans from World War II conflicts [24]. However, the nature and severity of the injuries were not defined. Pooled analysis confirmed that TI was linked to an increased risk of CHD-related mortality (risk ratio 1.57 : 95% CI 1.35–1.83; *I*^2^ = 0%, *p* = 0.77: *p* < 0.0001) (Figure 3). In the only cohort study of combat versus no combat (without significant TI) Schlenger (moderate ROB) did not observe a significant difference in CHD-related (3.02% vs. 2.33%) deaths among US Vietnam combat versus noncombat veterans [22].

### 3.5. Myocardial Infarction and CHD

The risk of CHD or myocardial infarction was reported in ten studies (Tables 1 and 2). Four studies (two cohort and two cross sectional) reported outcome data following TI; two reported an increased risk [9, 24], whilst two were neutral [19, 25]. In the first of the two studies reporting an increased risk, Yekutiel and colleagues observed a higher risk of CHD among 53 lower limb amputees wounded from 1948 to 1973 versus 159 age-matched healthy controls [26]. In the second study, Stewart and colleagues reported a significantly higher risk of CHD risk among combatants surviving very serious TI versus the age-matched general population [27]. Furthermore, each five-point increase in the Injury Severity Score was linked to a 13% increase in the adjusted risk of CHD (Hazard ratio 1.13; 95% CI, 1.03–1.25; *p* = 0.01) [27]. There were six studies (one cohort and five cross sectional) that reported on CHD risk following combat exposure without TI. An increased risk with combat was observed in three studies (two low and one moderate ROB) [28–30], with no significant difference noted in another three (one low, one moderate and one high ROB) [31–33].

### 3.6. Stroke

Seven studies reported stroke outcomes (Tables 1 and 2), but these were predominantly cross sectional studies and possessed a significant ROB. In the only cohort study of combatants following TI (moderate ROB) there was no reported difference in stroke rates between veterans with and without proximal amputation (three versus two strokes, respectively) [19]. Among the population with combat exposure without TI five studies were identified; three of these studies reported an increased risk with combat versus non-combat controls (all cross sectional; one with low ROB, one moderate, one high) [28, 29, 31]. Two of the five studies reported no difference in risk (one cross sectional and one cohort; one low ROB and one moderate) [32, 33].

### 3.7. Aortic Aneurysm

Two cross sectional studies reported the risk of aortic aneurysms following TI. In one study, Vollmar et al. observed a higher prevalence of infrarenal aortic aneurysms (5.8% vs 1.1%) among veterans (*n* = 329) with above knee amputations, compared to deployed veterans without (*n* = 702) (high ROB) [34]. In the second study, there was no difference (4.4% vs. 4.0%) in ultrasound detected aortic aneurysms among veterans with lower limb amputation versus a control population of similar age (high ROB) [25].

### 3.8. Hypertension

19 studies reported the risk of hypertension (Tables 1 and 2). There were ten studies (eight cross sectional and two cohort) relating to TI. The risk of hypertension, was reported to be increased in five studies (one moderate and four high ROB) [27, 35–38], with another five studies reporting no influence of TI (one moderate and four high ROB) in systolic hypertension [19, 25, 26, 34, 39]. There were nine studies of combatants without TI (three cohort and six cross sectional), four of which found risk of hypertension to be higher following combat (two low, one moderate and one high ROB) [28, 29, 40, 41]; four studies found no effect of combat (two low, one moderate and one high ROB) [31–33, 42], whilst one study found combat was associated with a lower risk (moderate ROB) [30].

### 3.9. Cardiometabolic Risk Factors

#### 3.9.1. Diabetes

The risk of diabetes was reported in 15 studies. Among the studies relating to combat TI, four studies (one cohort and three cross sectional) reported a significantly higher risk associated with TI (one moderate and two high ROB) [26, 27, 37, 43] and four (two cohort and two cross sectional) reported no difference in risk between veterans with and without TI (two moderate and two high ROB) [19, 24, 25, 34]. Among the studies of noninjured combatants, two (one cohort) reported an increased risk [40, 44], three (all cross sectional) no difference (two low and one high ROB) [28, 31, 42] and two (one cohort and one cross sectional)found a lower risk of diabetes compared with nonexposed controls (both low ROB) [30, 32].

#### 3.9.2. Metabolic Syndrome

Only one cross sectional study reported metabolic syndrome as a specific outcome. Etjahed and colleagues observed a 2-fold higher risk of metabolic syndrome (Defined according to the ATP III Criteria) [45] among 235 veterans with bilateral traumatic lower limb amputation versus controls from the general population (high ROB) [46].

#### 3.9.3. Blood Lipid Levels

Comparative lipid levels and risk of hyperlipidaemia were reported in 11 studies. Among TI veterans two studies (cross sectional) reported an increased lipid profile compared with controls (high ROB) [37, 46]. There were six (two cohort and four cross sectional) studies that all reported no difference in lipid levels or risk of hyperlipidaemia among combatants with TI versus controls of TI (3 moderate and three high ROB) [19, 24–26, 34, 39]. There were three studies of uninjured combat veterans (one cohort and two cross sectional); one study [40] reported a higher risk (moderate ROB) and two studies found no differences in lipids levels of combatants versus controls (one moderate and one high ROB) [31, 44].

#### 3.9.4. Other Cardiovascular Risk Factors

Only one study examined carotid intimal thickness (CIMT), a known surrogate for subclinical atherosclerosis, among non-injured combat and noncombat veterans versus their civilian population [33]. CIMT was found to be higher among combat veterans (802.4 ± 182.2 *μ*m) compared with noncombat veterans (757.7 ± 164.1 *μ*m) even after adjustment for potential confounders (including age and race). However, there was no significant difference in carotid plaque burden after adjusting for confounders. There were no identified studies that examined the comparative measures of arterial stiffness or atrial fibrillation with traumatic injury or military combat.

#### 3.9.5. Strength of Evidence

Table 3 summarises the overall SOE relating to the effects of combat ± TI upon cardiovascular risk. The SOE supporting of a link between combat-related TI and an increased risk of cardiovascular death and CHD-related death is low. There is insufficient SOE linking combat-related TI (mainly lower limb amputations) to adverse cardiovascular outcome or risk factors. There is also insufficient evidence that combat exposure, in the absence of significant traumatic injury, is associated with an increase in adverse cardiovascular outcomes or cardiovascular risk.

## 4. Discussion

This is the first systematic review to examine the effects of combat exposure and TI on CVD-related mortality, as well as a wide range of cardiovascular risk factors. There is low SOE to support an association between combat-related TI and an increased risk of cardiovascular death and CHD-related mortality. There is insufficient evidence to support an association between combat-related TI and increased cardiovascular risk. Furthermore, there is insufficient evidence to support a link between combat exposure without trauma and adverse cardiovascular risk or outcomes.

A PubMed search identified one systematic review and one literature review relating to cardiovascular risk following traumatic amputation in the last 30 years [10, 11]. Robbins et al. [11] undertook a systematic review of combat and non-combat related amputations on the outcomes of CVD (four studies) [9, 19, 36, 47]) and cardiometabolic risk (two studies) [43, 48], as well as examining joint and phantom limb pain. Unlike our current review, their injured cohort included both civilian and military participants. The most recent single study included in their systematic review was published 17 years ago and a pooled analysis of objective clinical outcomes (CVD and CHD-related death) was not undertaken. Naschitz & Lenger [10] undertook a literature review that was also published 10 years ago and the most recent study included was published >30 years ago [39]. They suggested a number of potential aetiological factors that may be implicated in an increased cardiovascular risk following traumatic amputation. These included increased insulin resistance, psychological stress, high risk behaviour among exposed subjects and the effects of abnormal blood flow proximal to the amputation. Other risk factors remain largely speculative and further research with a need to examine the other types of combat related TI.

These two previous reviews highlight the need for further, more contemporaneous data from studies of participants in more recent military conflicts. There is also a need to more robustly examine clinical cardiovascular endpoints, such as cardiovascular death, and recognised cardiovascular risk factors. If traumatic amputation truly leads to an increased risk of CVD, then it would be expected that there would be a relatively higher burden and prevalence of established cardiovascular risk factors. Our current analysis did not find this to be the case. Based on the assimilated data within our meta-analysis, there is insufficient evidence, at present, to support a link between severe combat related TI and the development of diabetes, metabolic syndrome, hyperlipidaemia, hypertension or increased arterial stiffness. Whilst our pooled analysis did demonstrate a significant association between combat related TI and both CVD and CHD related death, these data are drawn from only two studies, with a moderate degree of bias, for each of these outcomes. Given these facts, coupled with the moderate heterogeneity of the studies and their findings, the strength of evidence to support a link remains low.

The decision to additionally examine the published data relating to military combat in the absence of significant trauma on cardiovascular risk and outcomes was important. This was done in order to better understand the additive risk associated with combat related injury. Pooled data identified a marginal, yet significantly lower relative risk of cardiovascular death among the combat versus control groups. This is likely to be explained by the fact that combatants were likely fitter and younger than that of the comparator population of non-combat veterans and civilians. There have been several recent publications suggesting a potential link between combat, in the absence of TI, and adverse cardiovascular outcomes [30–33, 41] and we were keen to more robustly assimilate the current evidence.

We deliberately excluded previous studies that examined cardiovascular outcomes among selected military populations including those with PTSD, isolated traumatic brain and spinal injuries. This was undertaken to reduce potential bias. PTSD can be triggered by a wide variety of adverse life events including military combat [49]. It has been consistently linked to an increased cardiovascular risk compared that of ‘non-exposed' individuals [49–53]. This relationship was further endorsed in a very recent meta-analysis of Iraq and Afghanistan war veterans in which a number of plausible mechanism were explored [54]. Traumatic brain and spinal injury have also been linked to adverse cardiovascular risk [55]. The studies included in our systematic review would have likely contained some individuals with these injuries as well as PTSD. However this would have represented a far lower proportion of cases than that of a selected study of these conditions. For example, whilst the burden of PTSD is influenced by the population studied it generally affects about 20% of combat veterans [49]. By exploring the wider context of combat and traumatic injury (hence multiple injury types) beyond that of lower limb amputation, which tended to be the main focus of previous studies we would be able to appreciate the cumulative effects of these exposures including the potentially positive (e.g., related to improved fitness for deployments etc.,) and negative (e.g., PTSD). This wider population inclusion is critical, given the wide range and complexity of traumatic injuries following recent armed conflicts.

This review has a number of important limitations that need to be acknowledged. There were a large number of cross sectional studies and the majority of cohort studies were retrospective. The majority of included studies had a significant ROB with variable, and in several cases no, adjustment for important confounders. Many of the studies did not report effect estimates with confidence intervals and there was inconsistent reporting of the participant demographics. Although publication bias and selective outcome reporting is a major concern, we failed to identify any unpublished studies (on reviewing the grey literature) that would support this concern. One considerable source of potential bias relates to the large heterogeneity between differing military conflicts in terms of obvious differences in the type, intensity and duration of combat exposure (Vietnam vs. Iraq/Afghanistan Wars). Finally, this review included a large number of studies consisting of variable control groups and relating to historical conflicts that occurred more than 40 years ago, which raises some concern about the reliability of diagnoses and outcome reporting.

The limitations we have identified within the existing literature highlight the need for prospective cohort studies of combat veterans who were engaged in recent armed conflicts. These future studies should be designed in such a way that combat veterans with TI are compared with an age matched control population, without known cardiovascular disease, that were deployed to the same operations and at a similar time are followed up and reviewed to examine their relative cardiovascular risk profiles, as well as their psychological health. Addressing the evidence deficits identified above is the focus of the ongoing ADVANCE study, which is in the final phases of its baseline recruitment [56].

In conclusion, there is insufficient data to either support or refute an association between combat or combat related TI and either CVD or an increased burden of cardiovascular risk factors. There is a weak SOE in support of a link between severe combat related TI and CVD and CHD-related death. There is a need for further data from well conducted prospective cohort studies following recent combat operations.

## Figures and Tables

**Table 1:** Description of individual studies and their outcomes and findings.

Author year	Population	Numbers and type of exposure	Study design	Age in years	Male, % in combat group	Follow up	Outcomes	Key finding/covariate adjustment
*Combat + traumatic injury*								
Hrubec and Ryder 1980 [9]	US military WWII (1944–45) veterans	3890 proximal amputees	Retrospective cohort	>80% <30 years old at time of injury	100%	>30 years	All-cause and disease specific mortality	↑ adjusted all-cause (RR : 1.36 : 1.25–1.48) CVD (RR : 1.58 : 1.40–1.79) and CHD related death (RR : 1.56 : 1.36–1.79) among proximal amputees vs. injured. ↑ risk of all-cause (1.29 : 1.18–1.41), CVD (1.44 : 1.26–1.64) and CHD (1.45 : 1.24–1.68) death among proximal vs distal amputees and vs general population.
		2917 distal amputees						
				3 groups age matched				
		3890 injured		Ages at analysis not provided				
		US population (age matched)						

Labouret et al. 1987 [35]	French veterans	106 with combat related amputation (49 AKA)	Cross-sectional	Compared by age decades from 40–89 years	100%	>15 years	Systolic and diastolic blood pressure	Higher unadjusted prevalence of systolic (not diastolic) HTN in the amputees vs controls (56% vs. 29%; *p* < 0.02) and significant for each age decade comparison.
	WWI (1914) *n* = 23	184 age matched controls without HTN						
	WWII (1939) *n* = 67							
	Other *n* = 16							

Rose et al. 1987 [36]	US Vietnam War veterans	19 AKA	Cross-sectional	20–22 at injury and 35–36 years at analysis	100%	≥;15 years	Insulin response to glucose infusion	↑ unadjusted rate of HTN (10/19) in amputees vs controls (1/12; *p* < 0.05); no difference lipid levels.
		12 age matched controls						

Vollmar et al. 1989 [34]	German WWII (1939–1945) veterans	329 veterans with AKA	Cross-sectional	67.2 years AKA	100%	43.8 years from injury	Ultrasound diagnosis of infrarenal abdominal aortic aneurysms	↑ AKA in amputees vs controls (5.8% vs. 1.1%); no differences in risk of HTN, hyperlipidemia and DM (comparative data not reported)
		702 nonamputee veterans						
				68.1 years controls with comparable burden of CVD risk factors				

Yekutiel et al. 1989 [26]	Israeli War Veterans wars (1948–9, 1956, 1967, 1973)	53 traumatic lower limb amputees	Cross-sectional	57.2 years	100%	>20 years from injury	Hypertension, CHD and DM	↑ unadjusted prevalence of CHD in amputees vs controls (32.1% vs. 18.2%; *p* < 0.01) and DM (22.6% vs. 9.4%); no difference in HTN (35.8% vs. 35.2%)
		159 age and sex-matched controls						

Lorenz et al. 1994 [25]	German population conflicts not stated	226 veterans with traumatic lower limb amputations	Cross-sectional	Age not reported (short report)	Not reported	Unreported but >1 year	Ultrasound diagnosis of abdominal aortic aneurysms	No difference in prevalence of aortic aneurysms among amputees (4.4%) vs controls (4%). No difference in risk of hypertension, diabetes or hyperlipidemia.
		199 controls						

Peles et al. 1995 [43]	Israel defence force veterans 1948–1974	52 Amputees	Cross-sectional	Amputees 52 years controls 53 years	100%	33 years after injury	Insulin resistance and autonomic function	Age adjusted ↑ in insulin levels among amputees vs controls; No unadjusted difference in glucose, lipids and blood pressure
		53 nonmilitary controls						

Modan et al. 1998 [19]	Israeli army wounded 1948–1974	Cohort 1 201 veterans + traumatic lower limb amputation 1832 general US population	Retrospective cohort study	50% <40 years	100%	24-year	All-cause CVD and non CVD mortality	Two fold ↑ (amputees vs. controls) in unadjusted risk of all-cause (21.9% vs. 12.1% *p* < 0.001 among older) and CVD-related death (8.9% vs. 3.8%,*p* < 0.001).
		Cohort 2 101 amputees 96 controls (matched by age and ethnicity)	Cross-sectional					
							CV risk factors	Cohort 2 ↑ plasma insulin levels (2 hour post oral glucose load) in amputees; No differences in unadjusted CHD (19.8% vs. 16.7%), cerebrovascular disease (3.0% vs. 5.2%), obesity, DM, HTN (43.6% vs. 35.4%), hyperlipidemia (37.6% vs. 30.2%)

Shahriar et al. 2009 [37]	Iranian wars	327 bilateral lower limb amputees	Cross-sectional	42 years at analysis with age of 20.6 years at injury control group age not reported	100%	Mean 22.3	Obesity and CVD risk factors	↑ unadjusted risk of HTN (28.5% vs. 20.4%: *p* < 0.05), total and LDL cholesterol (*P* < 0.05) obesity (31.8% vs. 22.3%) and smoking (31.8% vs. 22.3%; *p* < 0.05) versus control
		Iranian general population (demographics undefined) [5]						

Kunnas et al. 2011 [24]	Finnish Military WWII veterans	102 injured combat veterans	Prospective cohort study	55 years	100%	28 years	CHD mortality	(↑ adjusted risk of CHD (HR 1.7 : 1.1–2.5; *p* = 0.02) death among injured/wounded vs control. No difference in total cholesterol or DM.
		565 non injured veterans						

Stewart et al. 2015 [27]	US Military Iraq and Afghanistan wars 2002–2011	3846 severe traumatic injuries	Retrospective cohort	25–29.2 years	≥98%	1.1–4.3 years	Armed Forces Medical Examiner System (AFMES) database of outcomes	Each 5-point ↑in the ISS linked to a 6%, 13% and 13% ↑ in the adjusted risk of HTN (OR 1.06; 1.02–1.09; *P* = 0.003), CAD (1.13; 95% CI 1.03–1.25; *P* = 0.01), DM (1.13; 1.04–1.23; *P* = 0.003). ↑ Risk versus control population
		Millennium cohort [30, 41]						

Ejtahed et al.2017 [46]	Iran veterans of Iran-Iraq War	235 veterans with bilateral traumatic lower limb amputations vs general population	Cross-sectional	31.5 years at injury and 52 years at follow up	100%	32.1 years form injury	Metabolic syndrome	2-fold ↑ in metabolic syndrome, including HTN, insulin levels, hyperlipidemia and obesity (amputees (62.1%) vs general Iranian population (27.5% )
				Age for comparator not reported				

*Uninjured combat* ^‡^								
Bullman et al. 1990 [20]	US Vietnam War veterans	6668 high-combat veterans deaths	Retrospective cohort	Similar ages in both groups	100%	Median follow up >5 years	ICD8 8 codes	↓ in proportionate CVD mortality vs control group (mortality ratio 0.93 : 0.88–0.98).
		27917 low combat veteran deaths						

O'Toole et al. 1996 [40]	Australian Vietnam War veterans	641 army veterans (10.8% injured) vs age-sex matched population expected	Cross-sectional	29.5 years at military discharge	100%	>15 years	Self-reported physical health status	↑ adjusted risk of HTN (RR 2.17 : 1.71–2.62), DM (2.71 : 1.32–4.09) and lipids (2.73 : 1.94–3.52); CVD (RR 1.98 : 0.52–2.33) not significant. No relationship between increasing combat burden to any CVD outcomes or risk factors.

MacFarlane et al. 2000 [21]	UK Military veterans of Gulf War I (1990–91)	53416 war veterans	Retrospective cohort	71.5% <30 years at study enrolment	97.7%	8 years	Multiple	No significant difference in all-cause (MRR 1.05 : 0.91–1.21) and CVD mortality (0.74 : 0.49–1.12) among deployed vs nondeployed veterans mortality.
		53450 nondeployed military						

Eisen et al. 2005 [42]	US Military Gulf War (1991)	1061deployed war veterans	Cross-sectional	30.9 years deployed	78% in both groups	10 years	Physical health and QOL	No significant difference in adjusted risk of DM (1.52 : 0.81–2.85) or hypertension (0.90 : 0.60–1.33).
		1128 nondeployed		32.6 years non deployed^∗^				

Granado et al. 2009 [41]	US Military (2001–2003) (25% Iraq and Afghanistan)	4385 combat	Prospective cohort	Not reported	74.8–86%	2.7 years	SF-36 questionnaire arterial hypertension	↑ adjusted incidence of HTN among multiple combat veterans vs. nondeployers (OR 1.33 : 1.07–1.65:*p* < 0.05).
		4444 deployed noncombat		But grouped by birth^∗^ decades				
		27232 nondeployed						

Kang et al. 2009 [28]	US Gulf War (1991) veterans	6111 war veterans	Cross-sectional analysis of prospective cohort	31.5 years for war veterans	79.9% active 78.2% control	14 years	Health questionnaires	↑ adjusted self-reported prevalence of HTN (RR 1.11 : 1.04–1.19), stroke (RR 1.32 : 1.14–1.52), CHD (RR 1.22 : 1.08–1.39) and obesity. No significant difference in DM (RR 1.11 : 0.99–1.25).
		3859 veterans not deployed to Persian Gulf						
				33.6 years for control (in 1991) ^∗^				

Johnson et al. 2010 [33]	US Veterans World War II 40.6% (1941–1945), Korean War 34.6% (1950–1953) Vietnam Conflict 16.8% (1961–1975)	1178 combat (13.1% veterans) 2127 noncombat (deployed) veterans	Prospective cohort	19–20 years at enrolment	100%	36 years after military entry	Carotid intima-media thickness (CIMT) and carotid plaque	↑ age-adjusted CIMT in combat veterans (Risk difference 12.79 *µ*m : 0.72–24.86) noncombat veterans. No significant difference in carotid plaque noted.
				57.3 years combat veterans				
		2,042 nonmilitary						
				51.8 years non veterans				
				54.1 years non-combat veterans^∗^				

Johnson et al. 2010 [44]	US Veterans World War II 40.6% (1941–1945), Korean War 34.6% (1950–1953) Vietnam Conflict 16.8% (1961–1975)	1178 combat veterans (13.1% injured)	Prospective cohort	19–20 years at enrolment	100%	36 years after military entry	Myocardial infarction unstable angina or CHD-related death	No significant differences in adjusted CHD between combat (13.2%) and noncombat veterans (11.3%), and nonveterans (11.6%); similar ischaemic stroke risk (7.76% vs. 5.22% vs. 6.43%). ↑ prevalence of DM combat vs noncombat but no significant difference in HTN, lipid profiles.
				57.3 combat veterans				
		2127 noncombat (deployed) veterans						
				51.8 non veterans				
		2,042 nonmilitary		54.1 non-combat veterans^∗^				

Crum-Cianflone et al. 2014 [30]	US Military Iraq and Afghanistan wars 2001–2009	12280 deployed combat	Prospective cohort	34.4 years at baseline and mean age at CHD diagnosis 43.1 years (comparative ages not reported)	84.4%	5.6 years	Coronary heart disease	Combatants ↑ adjusted (age, sex, race) risk of CHD (OR 1.63 : 1.11–2.40) vs deployed noncombat servicemen but ↓ unadjusted risk of DM and hypertension.
		10602 deployed noncombat						
		37143 nondeployed military						

Schlenger et al. 2015 [22]	US Vietnam War veterans	1632 theatre veterans	Retrospective cohort	41.5 years theatre veterans	>95%	>10 years	ICD codes for causes of death	No significant difference in all cause (16.79% vs. 16.61%), CVD (5.23% vs. 3.81%) or CHD-related (3.02% vs. 2.33%) deaths.
		716 Era (noncombat) veteran controls		40.9 years control				

Barth et al. 2016 [23]	UK Gulf War (1991)	621901 Gulf War veterans 746247 noncombat veterans	Retrospective Cohort	28 years – war veterans	93% active	13.6 years	All cause and disease specific mortality (ICD-9)	No difference in adjusted CVD mortality among Gulf War vs noncombat veterans (0.99 : 0.093–1.05) but ↓ all-cause mortality (RR 0.97 : 0.95%–0.99%). ↓ risk of all cause (RR 0.49 : 0.48–0.50) and CVD (RR 0.43 : 0.42–0.45) related mortality in Gulf War veterans vs. US population.
				30 years – noncombat veterans^∗^	86.7% control^∗^			
		US general population			Significant			

Sheffler et al. 2016 [32]	US Vietnam War veterans 1959–1973	107 combat veterans	Cross-sectional	45.4 years – combat	100%	10 years	Multiple health outcomes	↓ adjusted (OR 0.25 : 0.09–0.63; *p* = 0.003) rate of diabetes among noncombat servicemen. No difference in unadjusted CHD, hypertension, heart attacks or stroke.
		620 noncombat controls		46.0 years – noncombat				

Thomas et al. 2017 [31]	US Military veterans Vietnam war (43.6%)	564 combat veterans (29.2% injured)	Cross-sectional	59.0 years – combat	87.6–93%	>20 years	Validated health questionnaires	↑ adjusted risk of stroke (OR 1.38 : 1.03–3.33); no difference in adjusted risk of heart attacks, high cholesterol HTN and other heart disease.
				61.3 years – noncombat^∗^				
		916 noncombat veterans						

Hinojosa 2018 [29]	US Military Iraq and Afghanistan Wars 2012–2015	14932 combat veterans	Cross-sectional	56.1 years – veterans 48.8 years – control^∗^	66.3% in military group vs 42% in nonmilitary controls^∗^	>1 year	CVD outcomes	↑ adjusted prevalence of HTN in veterans (OR 1.49 : 1.23–1.81), CHD (OR 1.55 : 1.0–2.40), and heart attacks (2.26 : 1.41–3.62); ↑ rates of stroke among male only veterans (OR 3.32 : 2.03–5.47).
		135135 civilians						

CHD, coronary heart disease; DM, diabetes mellitus; CVD, cardiovascular disease; HTN, hypertension, Results presented in brackets as odds ratio, relative risk and 95% confidence intervals unless stated; CHD, coronary heart disease; DM, diabetes mellitus; CVD cardiovascular disease; HTN, hypertension; AKA, above knee amputation; ISS, injury severity Score. Results presented in brackets as odds ratio (OR), relative risk (RR), mortality rate ratio (MRR), hazard ratio (HR) and 95% confidence intervals unless stated; ^‡^refers to studies where proportion with traumatic injury <50%. ^∗^Detailed demographics for this population either not fully defined or disclosed.

**Table 2:** Relative outcomes investigated for included studies and their direction of findings.

	Year	Cardiovascular mortality	CHD-mortality	CHD/MI	Stroke	Abdominal aortic aneurysms	Carotid Intimal thickness	Diabetes mellitus	HTN	Metabolic syndrome	Increased lipid profile
*Combat + Traumatic injury*											
Hrubec and Ryder [9]	1980	↑	↑	-	-	-	-	-	-	-	-
Labouret et al. [35]	1983	-	-	-	-	-	-	-	↑	-	-
Rose et al. [36]	1987	-	-	-	-	-	-	-	↑	-	↔
Vollmar et al. [34]	1989	-	-	-	-	↑	-	↔	↔	-	↔
Yekutiel et al. [26]	1989	-	-	↑	-	-	-	↑	↔	-	-
Lorenz et al. [25]	1994	-	-	↔	↔	↔	-	↔	↔	-	↔
Peles et al. [43]	1995	-	-	-	-	-	-	↑	↔	-	↔
Modan et al. [19]	1998	↑		↔	↔	-	-	↔	↔	-	↔
Kunnas et al. [24]	2011	-	↑	-	-	-	-	↔	-	-	↔
Shariar et al. [37]	2009	-	-	-	-	-	-	↑	↑	-	↑
Stewart et al. [27]	2015	-	–	↑	-	-	-	↑	↑	-	-
Ejtahed et al. [46]	2017	-	-	-	-	-	-	-	↑	↑	↑

*Combat only*											
Bullman et al. [20]	1990	↓	-	-	-	-	-	-	-	-	-
O'Toole et al. [40]	1996	-	-	-	-	-	-	↑	↑	-	↑
MacFarlane et al. [21]	2000	↔	-	-	-	-	-	-	-	-	-
Eisen et al. [42]	2005	-	-	-	-	-	-	↔	↔	-	-
Granado et al. [41]	2009	-	-						↑	-	-
Kang et al. [28]	2009	-	-	↑	↑	-	-	↔	↑	-	-
Johnson et al. [33]	2010	-	-	-	-	-	↑			-	-
Johnson et al. [44]	2010	-	-	↔	↔	-	-	↑	↔	-	↔
Crum-Cianflone et al. [30]	2014	-	-	↑	-	-	-	↓	↓	-	-
Schlenger et al. [22]	2015	↔	↔	-	-	-	-	-	-	-	-
Barth et al. [23]	2016	↔	-	-	-	-	-	-	-	-	-
Sheffler et al. [32]	2016	-	-	↔	↔	-	-	↓	↔	-	-
Thomas et al. [31]	2017	-	-	↔	↑	-	-	↔	↔	-	↔
Hinojosa [29]	2018	-	-	↑	↑	-	-	-	↑	-	-

CHD, coronary heart disease; MI, myocardial infarction; HTN, hypertension; Number refer to study findings in terms of direction of effect in relation to combat/injury versus control population: ↑, significantly increased/positive; ↔, no significant difference; ↓ lower risk; - unreported.

**Table 3:** Summary of key clinical outcomes and strength of evidence.

Outcome	Study design/number of studies	Findings and direction	Overall strength of evidence
*Cardiovascular mortality*			
-CTI	Cohort 2, x-sectional 0	2 Positive 0 Negative	Moderate ROB; low SOE
-Combat only	Cohort 4, x-sectional 0	3 Neutral; 1 Negative (lower risk in combat vs control)	Moderate ROB; insufficient SOE

*CHD mortality*			
-CTI	Cohort 2, x-sectional 0	2 Positive	Moderate ROB; low SOE
-Combat only	Cohort 1, x-sectional 0	1 Negative (lower risk in combat vs control)	Moderate ROB; insufficient SOE

*CHD/myocardial infarction*			
-CTI	Cohort 1, x-sectional 3	2 Positive; 2 Neutral	High ROB, Insufficient SOE
-Combat only	Cohort 2, x-sectional 4	3 Positive: 3 Neutral	High ROB, Insufficient SOE

*Stroke*			
-CTI	Cohort 1, x-sectional 1	2 Neutral	High ROB, Insufficient SOE
-Combat only	Cohort 1, x-sectional 4	3 Positive and 2 Neutral	High ROB, Insufficient SOE

*Aortic aneurysm*			
-Combat only	Cohort 0, x sectional 2	2 Positive	High ROB, Insufficient SOE
-CTI	Cohort 0, x-sectional 0	No studies	Insufficient

*Carotid intimal thickness (CIMT)*			
-CTI	Cohort 0, x-sectional 0	No studies	Insufficient
-Combat only	Cohort 1, x-sectional 0	1 x Positive; No blinding; No difference in carotid plaque	Moderate ROB, Insufficient SOE

*Diabetes mellitus*			
-CTI	Cohort 2, x-sectional 6	4 Positive 4 Neutral	High ROB, Insufficient SOE
-Combat only	Cohort 2, x-sectional 5	2 Positive, 3 Neutral, 2 Negative	High ROB, Insufficient: high ROB

*Hypertension*			
-CTI	Cohort 1, x-sectional 9	5 Positive; 5 Neutral	High ROB, Insufficient SOE
-Combat only	Cohort 3, x-sectional: 6	4 Positive, 4 Neutral, 1 Negative	High ROB, Insufficient SOE

*Metabolic syndrome*	x-sectional: 1	CTI: 1 x Neutral	CTI: Insufficient
-CTI	Cohort 0, x-sectional 1	1 Positive	Very high ROB, Insufficient
-Combat only	No studies		Insufficient

*Hyperlipidaemia*			
-CTI	Cohort 2, x-sectional 6	2 Positive, 6 Neutral (variable or unreported case definition)	High ROB, insufficient SOE
-Combat only	Cohort 1, x-sectional 2	1 Positive, 2 Neutral	High ROB, insufficient SOE

CTI, combat related traumatic injury; CHD, coronary heart disease; SOE, strength of evidence; ROB, risk of bias; RR relative risk; OR, odds ratio; Negative studies refer to significantly lower risk of outcome vs control in active group.

## Data Availability

The data used to support the findings of this study are included within the article.

## Supplementary Materials

Supplementary Material Table 1: supplement study selection from search criteria. Table 2: supplement axis quality appraisal tool for included studies. Table 3: supplement ROBANS risk of bias assessment for included studies.
