# Supplementary material for: The Relationship between Military Combat and Cardiovascular Risk: A Systematic Review and Meta-Analysis
Source: Int J Vasc Med. 2019 Dec 22;2019:9849465. doi: 10.1155/2019/9849465 (PMC6942813; doi:10.1155/2019/9849465)
Supplement: Supplementary Material — Table 1: supplement study selection from search criteria. Table 2: supplement axis quality appraisal tool for included studies. Table 3: supplement ROBANS risk of bias assessment for included studies. [file 9849465.f1.docx]

**Supplementary Material**

**Table 1** Supplement Study selection from Search Criteria

| 1. Does the exposure consist of military combat including those experiencing traumatic injury during military operations   No -> STOP. Excluded (Not relevant to topic)  Yes -> Proceed to 2. |
| --- |
| 1. Is the study on human adults   No -> STOP. Excluded (Not relevant to topic)  Yes -> Proceed to 3 |
| 1. Is the article of any following study designs or publication types: Case report, Case series study, Review article, Opinion/Editorial, In-vitro and animal study   No -> Proceed to 4.  Yes-> STOP. Excluded (Excluded study design or publication type) |
| 1. Does the article report on any of the cardiovascular outcomes or Cardiovascular risk factors of interest   No -> STOP. Excluded (Not relevant to topic)  Yes -> Proceed to 5. |
| 1. Does the article include selected population with Post Traumatic Stress Disorder (PTSD)   No -> Proceed to 6  Yes -> STOP (selected and wrong population very high risk of bias) |
| 1. Does the article deal specifically with spinal cord injury   No -> Stop (wrong population very high risk of bias)  Yes-> Proceed to 7 |
| 1. Does the article deal with selected populations of prisoners of war, burns or other non-traumatic injury eg infection   No -> Proceed to 8  Yes-> Stop (wrong population very high risk of bias)Proceed to 7 |
| 1. Does the study include a comparator control group of non-combat exposed persons   No -> STOP (Excluded study design)  Yes-> Proceed |

**Table 2** Supplement Axis Quality Appraisal Tool for included studies

|  | **Year** | **1. Clear aims?** | **2. Design Appropriate to aims?** | **3. Sample size justified** | **4. Target and reference populations defined?** | **5. Sample population reflect target population?** | **6. Selection Process appropriate** | **7. Measures taken to address and categorise non response?** | **8. Risk factors and outcomes appropriate to aims?** | **9. Outcomes measured appropriately?** | **10. Clear definition of what was used to measure statistical significance** | **11. Statistical methods fully described?** | **12. Basic data adequately described?** | **13. No Response rate / inclusion bias concerns** | **14. Information about non-response data described** | **15. Results internally consistent** | **16. Result for analysis described in methods presented?** | **17. Discussions and conclusions justified; confounders considered** | **18. Limitations discussed sufficiently** | **19. Funding conflict of interest / unstated** | **20. Ethical approval stated?** | **Total** |
| --- | --- | --- | --- | --- | --- | --- | --- | --- | --- | --- | --- | --- | --- | --- | --- | --- | --- | --- | --- | --- | --- | --- |
| Hrubec (9) | 1980 | 1 | 1 | 0 | 1 | 1 | 1 | 1 | 1 | 1 | 1 | 1 | 0 | 0 | 0 | 1 | 1 | 1 | 1 | 1 | 0 | 15 |
| Labouret (35) | 1983 | 1 | 1 | 0 | 1 | 1 | 0 | 0 | 1 | 1 | 0 | 1 | 1 | 0 | 0 | 0 | 1 | 1 | 0 | 0 | 0 | 10 |
| Rose (36) | 1987 | 0 | 1 | 0 | 1 | 0 | 0 | 0 | 1 | 1 | 1 | 1 | 0 | 0 | 0 | 0 | 1 | 1 | 0 | 1 | 0 | 9 |
| Vollmar (34) | 1989 | 1 | 1 | 0 | 0 | 1 | 1 | 0 | 0 | 1 | 0 | 0 | 0 | 0 | 0 | 0 | 0 | 1 | 1 | 1 | 0 | 8 |
| Yekutiel (26) | 1989 | 1 | 1 | 0 | 1 | 0 | 1 | 0 | 1 | 1 | 0 | 0 | 0 | 0 | 0 | 1 | 1 | 1 | 0 | 1 | 0 | 10 |
| Bullman (20) | 1990 | 1 | 0 | 0 | 0 | 0 | 1 | 0 | 0 | 1 | 0 | 0 | 0 | 0 | 0 | 1 | 1 | 0 | 1 | 0 | 0 | 6 |
| Lorenz (25) | 1994 | 1 | 1 | 1 | 0 | 1 | 0 | 0 | 1 | 1 | 0 | 1 | 0 | 0 | 0 | 1 | 1 | 0 | 0 | 0 | 0 | 9 |
| Peles (43) | 1995 | 1 | 1 | 0 | 1 | 1 | 1 | 0 | 1 | 1 | 0 | 1 | 1 | 0 | 0 | 1 | 1 | 1 | 0 | 0 | 1 | 13 |
| O’Toole (40) | 1996 | 1 | 1 | 0 | 1 | 0 | 1 | 1 | 1 | 1 | 1 | 1 | 0 | 0 | 1 | 0 | 1 | 1 | 1 | 1 | 1 | 15 |
| Modan (19) | 1998 | 1 | 1 | 0 | 1 | 1 | 1 | 1 | 1 | 1 | 0 | 0 | 1 | 1 | 1 | 0 | 1 | 0 | 0 | 0 | 0 | 12 |
| MacFarlane (21) | 2000 | 0 | 1 | 0 | 1 | 1 | 1 | 1 | 1 | 1 | 1 | 0 | 1 | 0 | 0 | 0 | 0 | 1 | 0 | 0 | 0 | 10 |
| Eisen (58) | 2005 | 1 | 1 | 0 | 1 | 1 | 1 | 1 | 1 | 1 | 1 | 1 | 1 | 1 | 1 | 1 | 1 | 1 | 1 | 1 | 1 | 19 |
| Granado (41) | 2009 | 1 | 1 | 0 | 1 | 1 | 1 | 0 | 0 | 1 | 1 | 1 | 1 | 0 | 0 | 1 | 1 | 1 | 1 | 1 | 1 | 15 |
| Kang (28) | 2009 | 1 | 1 | 0 | 1 | 1 | 1 | 1 | 1 | 0 | 1 | 1 | 0 | 0 | 1 | 1 | 1 | 1 | 1 | 1 | 1 | 16 |
| Shariar (37) | 2009 | 1 | 1 | 0 | 0 | 1 | 0 | 0 | 1 | 1 | 1 | 0 | 0 | 0 | 0 | 0 | 0 | 0 | 0 | 0 | 0 | 6 |
| Johnson (59) | 2010 | 1 | 1 | 0 | 1 | 1 | 0 | 0 | 1 | 1 | 1 | 1 | 1 | 0 | 0 | 1 | 1 | 1 | 1 | 1 | 0 | 14 |
| Johnson.(44) | 2010 | 1 | 1 | 0 | 1 | 1 | 0 | 0 | 1 | 1 | 1 | 1 | 1 | 0 | 0 | 0 | 1 | 1 | 1 | 1 | 0 | 12 |
| Kunnas (24) | 2011 | 1 | 1 | 0 | 1 | 1 | 1 | 0 | 1 | 1 | 0 | 1 | 0 | 0 | 0 | 1 | 1 | 1 | 0 | 0 | 0 | 11 |
| Crum-Cianflone (30) | 2014 | 1 | 1 | 0 | 1 | 1 | 1 | 0 | 1 | 1 | 1 | 1 | 1 | 1 | 1 | 1 | 1 | 1 | 1 | 0 | 1 | 17 |
| Schlenger (22) | 2015 | 1 | 1 | 0 | 1 | 1 | 1 | 0 | 1 | 1 | 0 | 1 | 1 | 0 | 0 | 0 | 0 | 1 | 1 | 1 | 0 | 12 |
| Stewart | 2015 | 1 | 1 | 0 | 1 | 0 | 1 | 1 | 1 | 1 | 1 | 1 | 1 | 0 | 0 | 1 | 1 | 1 | 1 | 1 | 1 | 16 |
| Barth (23) | 2016 | 1 | 0 | 1 | 1 | 0 | 1 | 0 | 1 | 1 | 1 | 1 | 1 | 0 | 1 | 1 | 1 | 1 | 1 | 1 | 1 | 16 |
| Sheffler (32) | 2016 | 1 | 1 | 0 | 1 | 1 | 1 | 0 | 1 | 0 | 0 | 1 | 1 | 0 | 1 | 1 | 1 | 1 | 1 | 0 | 1 | 14 |
| Ejtahed (46) | 2017 | 1 | 1 | 0 | 0 | 1 | 1 | 0 | 1 | 1 | 1 | 1 | 0 | 0 | 1 | 0 | 0 | 1 | 0 | 1 | 1 | 12 |
| Thomas (31) | 2017 | 1 | 1 | 0 | 1 | 1 | 0 | 0 | 1 | 0 | 0 | 1 | 1 | 0 | 0 | 1 | 1 | 1 | 1 | 1 | 1 | 13 |
| Hinojosa (29) | 2018 | 1 | 1 | 0 | 1 | 1 | 1 | 0 | 1 | 0 | 0 | 1 | 1 | 0 | 1 | 1 | 1 | 1 | 1 | 1 | 0 | 14 |

**Table 3** Supplement ROBANS Risk of Bias assessment for included studies

|  | **Study year** | **Selection of participants** | **Confounding Variables** | **Exposure Measurement** | **Blinding of Outcome Assessment** | **Incomplete Outcome data** | **Selective Outcome reporting** | **Overall Bias Risk (0-6)** |
| --- | --- | --- | --- | --- | --- | --- | --- | --- |
| Hrubec (9) | 1980 | 0 | 0 | 0 | 0 | 0 | 0 | 0 |
| Labouret (35) | 1983 | 1 | 1 | 1 | 1 | 0 | 0 | 4 |
| Rose (36) | 1987 | 1 | 1 | 0 | 1 | 1 | 0 | 4 |
| Vollmar (34) | 1989 | 1 | 1 | 0 | 0 | 1 | 1 | 4 |
| Yekutiel (26) | 1989 | 1 | 1 | 0 | 1 | 0 | 0 | 3 |
| Bullman (25) | 1990 | 1 | 1 | 1 | 0 | 1 | 0 | 4 |
| Lorenz (20) | 1994 | 1 | 1 | 1 | 1 | 0 | 0 | 4 |
| Peles (43) | 1995 | 1 | 1 | 0 | 1 | 0 | 0 | 3 |
| O’Toole (40) | 1996 | 1 | 0 | 1 | 0 | 0 | 0 | 2 |
| Modan (19) | 1998 | 1 | 0 | 0 | 1 | 0 | 0 | 2 |
| MacFarlane (21) | 2000 | 0 | 0 | 0 | 0 | 0 | 0 | 0 |
| Eisen (58) | 2005 | 0 | 0 | 0 | 0 | 0 | 0 | 0 |
| Granado (41) | 2009 | 0 | 0 | 0 | 0 | 0 | 0 | 0 |
| Kang (28) | 2009 | 0 | 0 | 0 | 0 | 0 | 0 | 0 |
| Shariar (37) | 2009 | 0 | 1 | 0 | 1 | 1 | 1 | 4 |
| Johnson (59) | 2010 | 1 | 0 | 0 | 0 | 0 | 1 | 2 |
| Johnson.(44) | 2010 | 0 | 0 | 0 | 1 | 0 | 1 | 2 |
| Kunnas (24) | 2011 | 1 | 1 | 0 | 0 | 0 | 0 | 2 |
| Crum-Cianflone (30) | 2014 | 0 | 0 | 0 | 0 | 0 | 0 | 0 |
| Schlenger (22) | 2015 | 0 | 0 | 0 | 0 | 1 | 0 | 1 |
| Stewart | 2015 | 1 | 0 | 0 | 0 | 0 | 0 | 1 |
| Barth (23) | 2016 | 1 | 1 | 1 | 0 | 0 | 0 | 3 |
| Sheffler (32) | 2016 | 0 | 0 | 0 | 0 | 0 | 0 | 0 |
| Ejtahed (46) | 2017 | 0 | 1 | 1 | 1 | 1 | 1 | 5 |
| Thomas (31) | 2017 | 1 | 1 | 1 | 0 | 0 | 0 | 3 |
| Hinojosa (29) | 2018 | 0 | 1 | 1 | 0 | 0 | 0 | 2 |
| Risk of bias rated as low=0, high =1 and – means results unclear | | | | | | | | |
